# Suggestions in Reference to the Study of the Philosophy of the Human Mind

**Published:** 1855-10-01

**Authors:** Thomas Hunt

**Affiliations:** To the Editor of the Psychological Journal.


### SUGGESTIONS IN REFERENCE TO THE STUDY OF THE PHILOSOPHY OF THE HUMAN MIND.

BY THOMAS HUNT, F.R.C.S.

*To the Editor of the Psychological Journal.*

I ALWAYS read the papers of the "Psychological Journal" with interest; and assuredly the objects which it embraces are second to none connected with medical science. An essay "On the Uses and Influence of Mental Philosophy," by Dr. Rae, which appeared in the number for July, has attracted my careful attention; and whilst I agree with the excellent remarks of the author on the importance of the study of man in the higher departments of his nature, I must beg permission to introduce the subject of this communication with some friendly criticisms on that paper.

In the first place, I cannot agree with Dr. Rae in the opinion that "the slow progression of mental science" has been due to the general preference of mankind for the study of physical rather than of intellectual science: neither can I assent to the belief that the prejudice against psychological investigations which exists in the majority of mankind has anything to do with the matter. On the contrary, the study of mental philosophy has so many charms for a mind at all capable of applying itself to these subjects, that one cannot see how the disinclination of the majority of mankind can have diminished the ardour of the student. But, waiving this question, I am disposed to think that in gathering up arguments in favour of the cultivation of mental science, Dr. Rae has inadvertently confounded the subject of mental philosophy with *the popular study of human nature* in its moral characteristics; otherwise he would scarcely have classed "the Jesuits," "the present Napoleon," or "the Abyssinian traveller, Bruce," amongst the students of mental philosophy. Nor would he have attributed "the movement that is now going on in the Church of England" to the "kind of philosophy taught in the Universities." It is, however, extremely difficult for any writer who eschews technicalities to make himself clearly understood when discoursing on the arcana of the human mind.

My object on the present occasion is to offer a few suggestions to those who may hereafter pursue this subject, which I trust may be useful in diminishing in some degree the obscurity in which it is involved.

First of all, it should be observed that the students of human nature are naturally and actually divided into three classes—the metaphysical, the moral, and the physical. The class of students who have attempted the elucidation of the metaphysical philosophy of the human mind have of late somewhat obscured the naturally indistinct outline which bounds the objects of their study by dignifying the science with the name of *moral* philosophy. The scope of metaphysical inquiry having no necessary connexion with morals (or the doctrines concerning virtue and vice), the study would be better described as your correspondent defines it—"mental philosophy;" or, as Dugald Stewart calls it, "the philosophy of the human mind."

I propose to show, that beyond its utility in improving the powers of the mind and sharpening the wits of the student, mental or moral philosophy, commonly called metaphysics, is a pursuit utterly useless, and incapable of any practical application either to *physics* or *morals;* and that if we would study human nature to any good purpose, we must leave these abstractions, and examine the mind not as an entity but as we find it palpably presented to our view in its *physical* and *moral* relations. Within these plain and homely limits, I am ready to grant, that (as Dr. Rae justly remarks) "the study of the human mind is one of the noblest and most important which can engage the attention of mankind." I will endeavour to define these limits by a more ample illustration of the subject; and regarding the writers on the human mind as distinctly divisible into the three classes above alluded to—the metaphysical, the popular, and the medical—I propose to make a few remarks on each.

1. The *metaphysical* writers study the human mind in the abstract. Overlooking or purposely neglecting both the moral and physical peculiarities of individuals, they confine their attention to those features of the mind which are common to the whole species. They regard man simply as a being susceptible of impressions, and the precise objects of their study are the natural order and phenomena of mental processes. They endeavour to ascertain how the mental machine works; they wish to analyse its susceptibilities, its powers, properties, or states; they ask how reason acquires its powers and performs its wonders; and they endeavour to trace back every mental phenomenon to its source. They study the mind partly by regarding its connexion with the external world, and partly by endeavouring, though, as we think, in vain, to examine the objects of their own consciousness; they thus undertake to discover the cause, origin, and history of ideas, sensations, emotions, and all purely intellectual processes. This is the end and aim of modern metaphysics. The ancient philosophers never attempted so hopeless a task as to explore the human mind. Aristotle was too much of a philosopher to undertake anything so utterly impracticable. The schoolmen, it is true, were fond of abstractions and intellectual speculations, but these all had a practical reference to the development of virtue; and their design seems rather to have been to arrange and define the objects of thought than to explore the mental faculties themselves. It was reserved to our modern philosophers not only to attempt the dissection of thought, but to essay the analysis of the elements of mind itself; and so much gravity, and pomp, and pretension has been thrown around the operations of this mental chemistry, as to fascinate and bewilder almost every accomplished mind which has lent attention to the scheme. There is scarcely a more humiliating fact in the intellectual history of man than that, for the last two centuries, many minds of the highest order should have been from one generation to another grappling with metaphysical abstractions which were to become the foundation, the measure, and the criteria of all human science, but which the result has shown to be utterly intangible and void. During this enlightened period, every accomplished *natural* philosopher who has made pretensions to discovery, has established a reputation which can never die. Bacon and Boyle, Newton and Davy, Watt and Jenner, are names which will be familiar to distant generations. *Their* discoveries will never be ignored by improvements in physical science. Every future new invention will be built upon the foundation already laid by *them*. But a principle of mortality appears to be inherent in every theory of *mental* philosophy which has, during the same period, obtained a fleeting hold upon the human mind. Every new speculator who has appeared on this stage has, in his turn, done little more than sweep away the fragile monuments of the past, leaving in their place an edifice equally unstable and unsound. The *Ethics* of Aristotle, having survived the ordeal of two thousand years, are even now in the hands of the Professors at Oxford and Cambridge; but where are the "Vibrations" of Hartley? In what school is inculcated the "Idealism" of Berkeley? Who regards with respect the "Materialism" of Priestly? In what hidden sepulchre are entombed the "Categories" of Kant?

The great error has consisted in attempting the investigation of mind by a method analogous to the chemical analysis of matter. The method of Aristotle partook rather of the synthetic than of the analytic. He gathered his knowledge of mind from the qualities of known objects related to mind. The modern philosophers set to work with the attribute of mind itself, as though they were separable and divisible like the atoms of matter.

It is no longer a mystery, therefore, that "the reputation of no modern metaphysician has continued with undiminished lustre through the revolutions of a century." The celebrated Essay of Locke has lost its charms. The names of Berkeley and Hartley, men of great worth and high attainments, are associated with ideas now regarded as preposterous. Hume and Reid, Stewart and Brown, names once highly esteemed amongst modern philosophers, will scarcely be mentioned in the next generation.

For what earthly purpose, then, shall we attempt to cultivate a (so called) science, which, after engaging the attention of highly accomplished minds for several generations, has not left us one single principle, one undisputed inch of ground on which we can set our foot? If it be alleged that metaphysical philosophy is the *gymnasium* of the youthful mind—that all things seem easy and simple to the man who has long contended with these remote abstractions, and that without such exercises the mind cannot attain to its normal development—my reply is, the giants of antiquity needed no such helps. Aristotle, Theophrastus, Hippocrates, Galen—all achieved their respective successes without entering the arena of modern philosophy. Let it be fully granted that these exercises do give strength and agility to the mind, that they enable it to discriminate more readily, to distinguish shades of difference between ideas and propositions which the vulgar would confound,—still, I maintain that all these advantages would be better secured, as well as more readily acquired, by the study of logic and mathematics. Young men who have neglected these first principles of reasoning and become bewildered on the enchanted ground of metaphysical abstraction have rarely distinguished themselves in after life. The mind is liable to become paralysed by attempting what is evidently beyond its strength, and the more homely studies which fit men for the duties and business of life become distasteful to the towering spirit, just as the panorama of Primrose Hill offers no attractions, the Peak of Snowden no wonders, to the practised aeronaut. A man who only *thinks* he can fly despises pedestrianism even while he walks.

2. The *moral* or *popular* writers on human nature have no sympathy with the metaphysicians. They see the mind of man through a different medium, and study it with other views. If our modern philosophers, who look at man only in the abstract, have scarcely shed a ray of light on the phenomena of mind, still less have they expounded the moral and social qualities of human nature. But it is here that the moralist takes his stand. He studies and portrays mankind just as he individually is, not in his essence or abstractions, but in his social relations and moral attributes. That this mode of investigating human nature (correctly speaking, the true *moral* philosophy) has been abundantly successful, is obvious to all. Our divines, historians, poets, novelists, and dramatic writers well understood their task; and they have earned a fame as imperishable as that of Bacon and Newton. The names of Addison, Johnson, and Shakspere, will be known when modern metaphysics and its professors are forgotten. It is to this popular study of human nature that we are indebted, and not to mental philosophy, for the influence exercised by the writers alluded to by Dr. Rae, who has evidently confounded these two departments of study, as unlike each other as commerce and classical literature.

3. But the *physical* study of man, in his mental development, the department of the physician, is that which most concerns the psychologist: and this brings me to the question, the importance of which first induced me to take up my pen:—Is it advisable, or desirable, that a student of medicine, intending to practise his profession, should give his mind to the study of metaphysics?

It is essential, certainly, that the physical relations of the mind should be most carefully studied. The healthful condition of both body and mind, especially in their mutual relationship to each other, must be observed and understood, before their morbid conditions can be apprehended. And in disease, whether of body or mind, who can exaggerate the importance of rightly estimating the reciprocal influence of both on each other? If it be the special business of the psychologist to ascertain and diagnose the physical causes of insanity, it is not less the duty of the physician, ay, and of the surgeon too, to mark well the influence of affections of the mind in producing disease of the body. Much has been written on the effects of mental hysteria in inducing simulations of disease; but the influence of mental emotion on the corporeal frame of both sexes is a subject which, though forming the staple commodity of novel writers, has perhaps scarcely attracted sufficiently the study of the profession. An accomplished girl of extraordinary personal attractions, and the heiress of a pretty fortune, fell in love with a lame cobbler of diminutive stature and repulsive physiognomy. Such things will happen. Her attachment was warmly returned; but the course of true love never did run smooth, and an impediment existed, besides the natural opposition of the parents to so preposterous a match. She was, and had been for some time (under the care of a celebrated oculist) suffering from severe pain in the orbit and globe of each eye, whenever she opened her eyes or attempted to use them. The disease was diagnosed "an hysterical intolerance (not of light but) of vision." The lameness of the cobbler was attributed to some strumous affection of the hip joint, and he had been treated accordingly, but with no benefit. One fine morning the couple contrived to accomplish a clandestine marriage. They entered the church respectively blind and maimed: he with his usual halt, walking with a crutch, she with a green shade over her brow, her eyes closed, and led by her lover to the chancel steps. The ceremony over, the cobbler recovered the use of his leg and threw away his crutch; the lady found her vision quite as marvellously restored, and from the time she made her egress from the church, suffered no pain or inconvenience in reading the smallest print. These I know to be facts, and, after the most rigid inquiry, I am satisfied there was no sham or imposture in either case. The diagnosis of the cobbler's lameness might have been erroneous, though it was made by a highly respectable surgeon. This case, and a hundred others which might be quoted, serve to show that when we cannot readily account for disease, it will be as well to inquire into the state of the mind. And this leads me further to insist that,

The *moral* study of human nature is highly desirable for the student of medicine; and in order to pursue this to an available extent, he must study mankind as Shakspere did—not metaphysically, overlooking the individual, nor by observing the conduct of an individual or a sect or a province, *only;* but by observing human nature in all its relations and phases, and, so far as is possible, at all times and seasons. Men and women, except on special occasions, are very apt to conceal their emotions, motives, and springs of action; but dramatic incidents and occasions, and the not less romantic events of real life and historic record, bring them out, and show how closely interwoven are the infirmities of mind and body. And so universally is mankind subject to moral aberration, folly, and vice, so weak in resolve, and when resolved, so indeterminate in action; so easily led into errors which he *sees* are wrong, so readily tempted to conduct which he knows he shall repent—or else reckless of consequences, unscrupulous and wicked — that every sensible writer on human nature, of whatever nation, or class, or date, has invariably portrayed mankind as *morally deranged.* Some have even excused his excesses on this very ground; but the common sense of mankind, as expressed in the criminal code of every civilized nation, has pronounced differently, and made man responsible for his crimes. For many generations the commission of crime was regarded as a sufficient ground of punishment. The physical condition of the brain, as concerned in perverting the moral sense, was seldom or never taken into the account. The subject has excited the most diligent attention of late years, and medical men are now in danger, not of overlooking the state of the brain in criminal lunatics, but of underrating the moral derangement which may exist, even in its most atrocious forms, when the brain is perfectly sound, and the individual consequently responsible. Now, the remedy for this liability to error is the diligent study of human nature in its *moral* aspects. The historical portions of the Bible, and the works of Shakspere, contain the best illustrations of this subject. Scott delighted to mingle the immoral and the insane affections so hazily together for the purpose of effect, that many of his most prominent characters throw little light on the question; but his subordinates for the most part make excellent studies.

I cannot conclude these remarks without pointing out a great deficiency in the *medical mind* of this country, arising from some error in education. I allude to the strange want of *logic* in the medical authors of the day. Every medical reviewer is constantly pointing out how this, that, and the other author is "reasoning in a circle," "begging the question," "reasoning on opinions as though they were facts," "assuming as facts things only probable," or "representing as facts, and reasoning on them, events which have never occurred;" and there are ten chances to one that, before the reviewer has finished his criticism, he falls into the same error himself. Now, all this is due, if in part to prejudice, yet chiefly to the want of mental discipline, and instruction and practice in the rules of logic in early life. As men *begin* to think when the mind is expanding into maturity, so they continue to think till their lives' end. If they get into early habits of vicious ratiocination, these are almost sure to become inveterate and incurable; some of the highest ornaments of the profession, so far as diligence, tact, and perseverance have brought them into note, are at this moment rendering themselves ridiculous and contemptible by their notoriously *false reasoning* about opinions, persons, and things. It is but charitable to suppose that the same defect (easily rectified in early life) may have plunged some hopeful aspirants into the slough of homœopathy.

It is certain, at all events, that a man who *cannot* reason well (for I am not alluding to dishonest reasoners), must find a limit to his fame and influence as he advances towards the higher degrees of professional reputation. Nothing can compensate for this radical defect. A want of classical knowledge may cripple his reading and limit his influence with the higher classes of society; but this will not clip his wings. A good surgeon is scarcely the better surgeon for classical lore, though he may the more adorn his profession: but no physician, nor even surgeon, can clearly understand his profession without a knowledge of the art of reasoning. *Mathematics*, though absolutely necessary for understanding the mechanism of the human frame, will not supply the want; for, although the mind requires the discipline of mathematical study to render it capable of understanding logic, yet, it must be remembered, medicine is not an exact science. It deals, and must deal, with probabilities as well as facts. Medical doctrines must for the most part be received, as Bishop Butler has shown the Christian doctrine must be embraced, not by absolute demonstration, but by a balance of probabilities. In the case of Christianity, the balance is, to every candid mind, overwhelming; but in medical matters the preponderance is very small, often where life and death are suspended in the scale. And, though our lenient laws make due allowance for a man who, with the best intentions, errs, yet it is easy to see how the life of a patient may be sacrificed to the misapprehension of a mind duly informed, but not duly skilled in balancing the weight of evidence.

---

